# *Pseudomonas aeruginosa* 4-Amino-4-Deoxychorismate Lyase: Spatial Conservation of an Active Site Tyrosine and Classification of Two Types of Enzyme

**DOI:** 10.1371/journal.pone.0024158

**Published:** 2011-09-15

**Authors:** Patrick E. F. O'Rourke, Thomas C. Eadsforth, Paul K. Fyfe, Sharon M. Shepherd, William N. Hunter

**Affiliations:** Division of Biological Chemistry and Drug Discovery, College of Life Sciences, University of Dundee, Dundee, United Kingdom; University of Glasgow, United Kingdom

## Abstract

4-Amino-4-deoxychorismate lyase (PabC) catalyzes the formation of 4-aminobenzoate, and release of pyruvate, during folate biosynthesis. This is an essential activity for the growth of Gram-negative bacteria, including important pathogens such as *Pseudomonas aeruginosa*. A high-resolution (1.75 Å) crystal structure of PabC from *P. aeruginosa* has been determined, and sequence-structure comparisons with orthologous structures are reported. Residues around the pyridoxal 5′-phosphate cofactor are highly conserved adding support to aspects of a mechanism generic for enzymes carrying that cofactor. However, we suggest that PabC can be classified into two groups depending upon whether an active site and structurally conserved tyrosine is provided from the polypeptide that mainly forms an active site or from the partner subunit in the dimeric assembly. We considered that the conserved tyrosine might indicate a direct role in catalysis: that of providing a proton to reduce the olefin moiety of substrate as pyruvate is released. A threonine had previously been suggested to fulfill such a role prior to our observation of the structurally conserved tyrosine. We have been unable to elucidate an experimentally determined structure of PabC in complex with ligands to inform on mechanism and substrate specificity. Therefore we constructed a computational model of the catalytic intermediate docked into the enzyme active site. The model suggests that the conserved tyrosine helps to create a hydrophobic wall on one side of the active site that provides important interactions to bind the catalytic intermediate. However, this residue does not appear to participate in interactions with the C atom that undergoes an *sp* ^2^ to *sp* ^3^ conversion as pyruvate is produced. The model and our comparisons rather support the hypothesis that an active site threonine hydroxyl contributes a proton used in the reduction of the substrate methylene to pyruvate methyl in the final stage of the mechanism.

## Introduction

**Figure 1:**
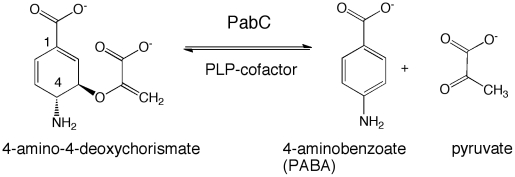
PabC catalyzes the conversion of 4-amino-4-deoxychorismate to 4-aminobenzoate and pyruvate.

Aminodeoxychorismate lyase catalyzes the formation of *para*-aminobenzoate (PABA) from 4-amino-4-deoxychorismate (Figure 1) as part of the biosynthetic route to folate [1] [2]. The enzyme, encoded by the *pabC* gene, is labeled as PabC.

In *Escherichia coli* and many other bacteria, PABA is synthesized in two steps starting from chorismate [3]. The first step produces 4-amino-4-deoxychorismate [4] by transferring ammonia, derived from glutamine, to chorismate. This reaction is performed by PABA synthase, a heterodimer of proteins encoded by the *pabA* and *pabB* genes [5]. 4-amino-4-deoxychorismate is then converted to PABA with the loss of pyruvic acid (Figure 1). The pyridoxyl 5′-phosphate (PLP) - dependent PabC catalyzes this second step [2] [3] [6] [7]. In the Gram-positive *Bacillus subtilis* a third chemical step is required for PABA biosynthesis. Chorismate is first converted to 2-amino-2-deoxyisochorismate before production of 4-amino-4-deoxychorismate, which is then converted to PABA and pyruvate by PabC [8].

PABA is required for the biosynthesis of folic acid and by extension also of essential metabolites such as thymidylate and methionine. In addition, PABA is required for biosynthesis of antibiotics such as candicidin [9] and certain *Streptomycetes* even possess additional copies of *pabC*, located in antibiotic production operons [10].

The inhibition of PabC would reduce PABA levels and so deplete the supply of folic acid available to the microorganism. Provided that the microbe cannot obtain sufficient PABA from the cellular environment or by other means these cells would be expected to cease growth and die. This is what is observed in *pabC* knockouts in *E. coli* [7], *Pseudomonas aeruginosa* [11], *Helicobacter pylori* [12] and *Acinetobacter baylyi* [13]. A few anti-microbial agents exert their effect through inhibition of folate biosynthesis. Sulphonamides for example deplete bacterial intracelluar folate levels by inhibiting PABA synthase and dihydropteroate synthetase [14]. Combining novel PabC inhibitors with sulphonamides or with other anti-folates such as the dihydrofolate reductase inhibitor trimethoprim, could produce a synergistic anti-microbial effect and new inhibitors of PabC would allow such a hypothesis to be tested.

X-ray crystal structures of PabC from *E. coli* [15], *Thermus thermophilus* [16] and *Legionella pneumophila* [Protein Data Bank (PDB) code 3lul] have been determined. PabC displays a similar overall fold to branched-chain amino acid transferases and D-amino acid transferases [15] [17] [18]. These aminotransferases have been investigated to identify potential inhibitors for example in support of research for treatments of neurodegenerative disease [19] [20] [21] [22]. Moreover, since PLP-dependent enzymes provide opportunities for the design of mechanism-based suicide inhibitors [17] this enzyme family appears an attractive one from the perspective of structure and mechanism-based drug discovery. The absence of the enzyme in humans and its essentiality in various microbes suggests that inhibition of PabC offers the possibility of new therapies targeting a range of microbial infections and structural studies provide useful data to assess the potential of this protein for such early stage drug discovery [23].

We have a particular interest in pathogens for which current treatments are unsatisfactory, for example Gram-negative bacteria such as *P. aeruginosa*, that are highly adaptable to their environment and which possess efficient and varied drug-resistance mechanisms. *P. aeruginosa* is often the cause of life-threatening infections in people with cystic fibrosis, burns victims and immuno-compromised individuals [24] [25]. We describe the construction of an efficient bacterial recombinant expression system, enzyme purification and crystallization protocols, and report the high-resolution crystal structure of PabC from *P. aeruginosa* (*Pa*PabC). Comparisons with previously determined PabC structures are presented, concentrating on active site cofactor interactions. Our structure-based comparisons identified the potential for a tyrosine to participate in catalysis and suggested that a re-evaluation of a published mechanism is warranted.

## Materials and Methods

### Cloning, protein purification and analytical ultracentrifugation

The *P. aeruginosa pabC* gene (locus tag: PA2964) was amplified from genomic DNA (American Type Culture Collection 47085, strain PAO1) by PCR using the following primers:

*Pa*PabC forward primer

5′-**CAT ATG** CTG GAC TGG GTC GAC-3′ (*Nde*I restriction site in bold)

*Pa*PabC reverse primer:

5′-**GGA TCC** TCA GAA ATC CAG GTC G-3′ (*Bam*HI restriction site in bold)

The PCR product was blunt-end ligated into a TOPO cloning vector (Invitrogen) then ligated into a modified pET15b expression vector (Novagen) that produces a hexahistidine-tag linked via a tobacco-etch virus (TEV) protease cleavage site. The resulting plasmid was heat-shock transformed into *E. coli* BL-21 (DE-3) GOLD cells (Novagen). Cultures were grown at 37°C in 2 L of Luria-Bertani medium supplemented with 100 µg mL^−1^ carbenicillin. Gene expression was induced, when the OD_600_ reached 0.6, by addition of 1 mM IPTG and growth continued for a further 16 hours at 18°C. Cells were harvested by centrifugation (3000× *g* at 4°C for 30 minutes), resuspended in 50 mM Tris-HCl, 200 mM NaCl, pH 7.5, and lysed by passage through a French pressure cell. Cell lysate was clarified by ultracentrifugation (40000× *g* at 4(C for 30(minutes) and the supernatant was passed through a 0.2 µm filter.

The lysate was loaded onto an immobilised metal ion affinity chromatography column (5 mL HisTrap HP, GE Healthcare) with the assistance of an FPLC system (Äkta Explorer, GE Healthcare). The hexahistidine-tagged protein was eluted from the column by a linear concentration gradient of imidazole and fractions were analysed by SDS-PAGE. Selected fractions were pooled and dialysed into 100 mM HEPES, 500 mM NaCl pH 7.5. Attempts to cleave the hexa-histidine tag by addition of TEV protease had produced large amounts of precipitate so it was decided to leave the purification tag in place. The protein was further purified by size exclusion gel chromatography (Superdex 200 26/60, GE Healthcare). This column had previously been calibrated with molecular weight standards, blue dextran (>2,000 kDa), thyroglobulin (669 kDa), ferritin (440 kDa), aldolase (158 kDa), conalbumin (75 kDa), ovalbumin (43 kDa), carbonic anhydrase (29.5 kDa), ribonuclease A (13.7 kDa) and aprotinin (6.5 kDa); (GE Healthcare; data not shown). Protein purity was assessed by SDS-PAGE, matrix assisted laser desorption/ionisation – time of flight mass spectrometry (MALDI-TOF MS) and electrospray ionisation mass spectrometry.

The protein concentration was determined spectrophotometrically using a theoretical extinction coefficient of 33,460 M^−1^ cm^−1^ (ProtParam, [26]).

### Crystallization, X-ray data collection, structure solution and refinement

*Pa*PabC was crystallized at 20°C by the hanging drop vapour diffusion method. The enzyme was concentrated to 33 mg mL^−1^ in 100 mM HEPES, 500 mM NaCl pH 7.5. Stock solutions of PLP (100 mM in DMSO) and PABA (200 mM in deionized water) were prepared and added to the PabC solution to give final concentrations of 0.1 mM PLP and 10 mM PABA. The mixture was left to incubate for 3 hrs then crystallization drops were assembled by mixing 1 µL of the protein∶ligand mixture with 1 µL of reservoir containing 10% (w/v) PEG 400, 1.8 M ammonium sulfate and 100 mM MES pH 6.5. Clumps of pale yellow, orthorhombic blocks grew over one week and a fragment of approximate dimensions 0.3 mm×0.2 mm×0.1 mm was used for data collection. Addition of cryoprotectant (25% v/v glycerol) was required to allow crystals to be mounted in a gaseous nitrogen cryostream at −173°C for diffraction experiments. Crystals were initially characterized using a copper anode X-ray generator (Rigaku 007) and an image plate detector (RAXIS IV^++^). The crystal was stored in liquid nitrogen for data collection at the European Synchrotron Radiation Facility (ESRF, Grenoble, France).

Diffraction data were collected on beamline ID29 at ESRF using a Quantum4 CCD detector. It proved beneficial to use the automated crystal annealing facility established on the station. Data were indexed and integrated using MOSFLM [27], and scaled with SCALA [28]. Crystallographic statistics are presented in Table 1. The search model for molecular replacement was prepared by generating a poly-alanine model of the *E. coli* PabC structure (PDB code 1et0, [15]) from which all waters and non-protein molecules had been removed. This model shares 35% sequence identity with *Pa*PabC. Molecular replacement calculations were performed using MOLREP [29].

**Table 1:** Crystallographic statistics.

Space group	*P* 2_1_2_1_2_1_
Unit cell dimensions: *a*, *b*, *c* (Å)	40.8, 66.8, 202.7
Resolution range (Å)	30.00 - 1.75
Unique reflections	56255
Completeness (%)	98.9 (98.5)^a^
*<I/*σ*(I)>*	10.7 (2.5)
Multiplicity	3.7 (3.3)
*R_merge_* b	7.8 (45.1)
No. of reflections	53372
*R_cryst_* c, *R_free_* d	16.7, 21.5
Protein residues	538
Ligands	2× PLP, 3 Cl^−^, 2 SO_4_^2−^, 2 glycerol, 3 di(hydroxyethyl)ether, 5 tetraethyleneglycol, 3 1,2-ethanediol & 474 waters
r.m.s. deviations from ideal geometry	
bond lengths (Å)	0.025
bond angles (°)	2.1
*B* values (Å^2^)	
From Wilson Plot	18.4
Mean *B* values	Chain A, main chain atoms: 15.1
	Chain A, side chain atoms: 18.2
	Chain B, main chain atoms: 18.5
	Chain B, side chain atoms: 20.7
	Waters: 30.8
	PLP: Chain A 15.8 Chain B 21.5
	Other ligands 36.3
Ramachandran favoured/allowed (%)	93.7/99.4 (3 outliers: Arg90, Chains A, B Leu89, Chain B)

a Values in parentheses refer to the highest resolution shell (1.84−1.75 Å).

b *R_merge_* = ∑*_hkl_∑_i_|*I*_i_*(*hkl*)−<I(*hkl*)>*|/*∑*_hkl_∑_i_* I*_i_*(*hkl*); where I*_i_*(*hkl*) is the intensity of the *i*th measurement of reflection *hkl* and <I(*hkl*)> is the mean value of I*_i_*(*hkl*) for all *i* measurements.

c *R_cryst_* = ∑*_hkl_*||*F_o_*|−|*F_c_*||/∑|*F_o_*|, where *F_o_* is the observed structure factor and *F_c_* is the calculated structure factor.

d *R_free_* is the same as *R_cryst_* except calculated with a subset, 5%, of data that are excluded from refinement calculations.

**Figure 2:**
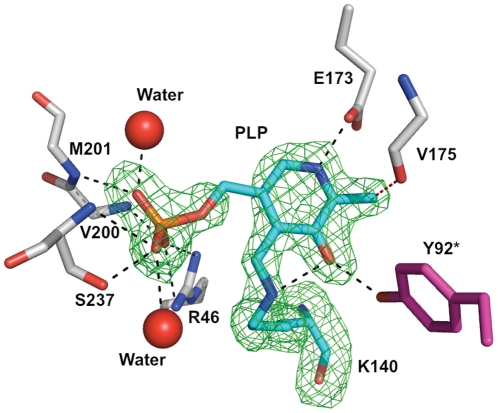
The omit map for PLP and Lys140 from subunit A. |(*F_o_*)-(*F_c_*)| difference density contoured at 3σ (green chicken wire). Selected hydrogen bonding associations between the protein, two water molecules (red spheres) and PLP are depicted as dashed lines. A * marks Tyr92 as contributed from subunit B. The side chain of Glu173 displays two rotamers with only one shown.

Refinement was performed in REFMAC5 [30] [31] and the model was inspected and manipulated in COOT [32]. Strict non-crystallographic symmetry restraints were employed in the early stages of the refinement and then removed. The presence of the PLP cofactor covalently bound to Lys140 was confirmed by calculating an omit electron density map (Figure 2). The N-terminal histidine tag was not visible in electron density maps. A number of ligands (tetraethylene glycol, 1,2-ethanediol, di(hydroxymethyl)ether, glycerol, chloride and sulfate ions) were assigned on the basis of the difference density and chemical environment, and refined satisfactorily. Both tetraethylene glycol and ethylene glycol are likely decomposition products of PEG400 used in crystallization, impurities or represent partially ordered molecules. MOLPROBITY [33] was used to assess model geometry, AREAIMOL [31] and the Protein Interfaces, Surfaces and Assemblies, EMBL server [http://www.ebi.ac.uk/msd-srv/prot_int/pistart.html; [34]] were used to calculate surface and interface areas. Figure 1 was prepared with CHEMDRAW (Adept Scientific), Figure 5 with ALINE [35] and the remainder using PyMOL [36] and ILLUSTRATOR (Adobe Systems Inc.).

### Amino acid sequence alignments and analysis

A search of the UniProt database with the Enzyme Commission number for PabC (4-amino-4-deoxychorismate lyase; 4.1.3.38) identified 368 entries. These were clustered such that sequences with a 90% or greater similarity to each other were grouped and a representative sequence identified. This reduced the list to 197 entries. Sequences consisting of more than 400 amino acids were removed and this further reduced the list to 181 entries. These were aligned using MUSCLE [37] and then inspected following pairwise alignment with *Pa*PabC. A conservative group of 129 putative PabC sequences was then identified following the removal of sequences that were fragmentary, labeled as other proteins or which displayed a sequence identity of less than or equal to 20%.

### Molecular modeling of the catalytic intermediate

A model of the substrate, 4-amino 4-deoxychorismate, was prepared using PRODRG [38] and placed into the active site using the molecular graphics program COOT [32] with the position of the bound pyridoxyl 5′ phosphate as a guide to allow formation of the catalytic intermediate. The active site was prepared for docking of this ligand using ICM pro (MolSoft L.L.C; http://www.molsoft.com/index.html) with the centre of the ligand-binding site defined by a cavity that contained the residues within 5 Å of the 4-amino 4-deoxychorismate. The top ten docking poses, as scored by ICM Pro software gave similar orientations in the active site and one was selected for discussion and display.

## Results and Discussion

### Overall structure

**Figure 3:**
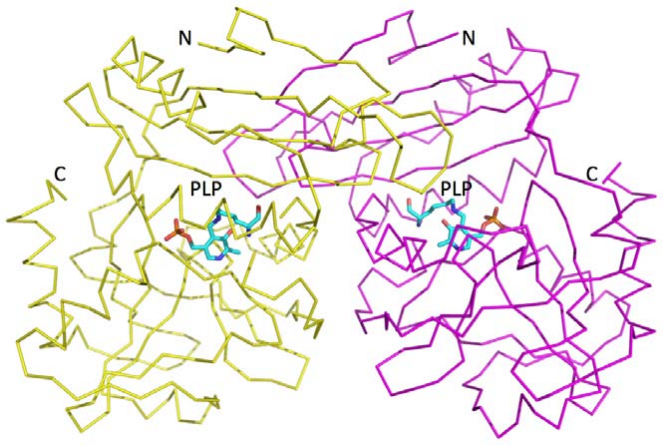
The *Pa*PabC dimer. Each subunit is depicted as a Cα trace (yellow and purple) with the PLP shown as stick model in a similar fashion to Figure 3. The N and C – terminal residue positions are labelled.

Size exclusion gel filtration, carried out during protein purification, indicated a single species of approximate mass 50 kDa that is commensurate with a homodimeric species. In the crystal structure two polypeptide chains, labelled A and chain B together constitute the asymmetric unit in *Pa*PabC (Figure 3), and the crystals have an estimated bulk solvent content of 45%. The polypeptide chains form a dimer (Figure 3) and, as described below, such oligomerization is required to create the enzyme active site. The presence of a *Pa*PabC dimer is consistent with previous studies since all PabC structures solved to date are dimeric or in the case of *T. thermophilus* PabC, a dimer-of-dimers is observed [16].

The *Pa*PabC subunits display a high degree of non-crystallographic symmetry. The rmsd following a least-squares fit of 270 Cα positions is 0.4 Å. It is therefore only necessary to detail one subunit and one active site.

**Figure 4:**
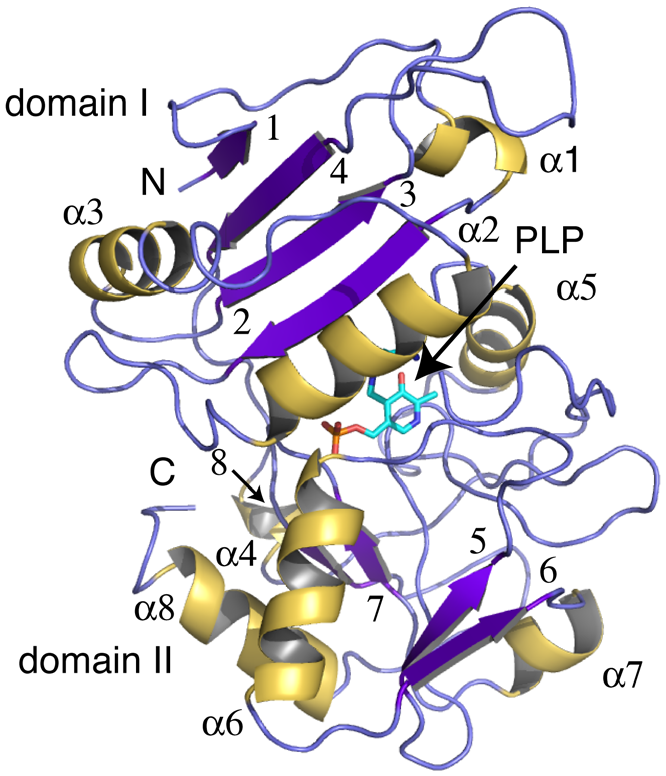
Ribbon diagram of the *Pa*PabC monomer (β-sheet is shown in purple, α-helix in yellow). Domain I is formed by α-helices 1–3 and the anti-parallel β-strands 1–4. Domain II is formed by α-helices 4–8 and β-strands 5–8.

The *Pa*PabC subunit is constructed from two domains (Figure 4). The N-terminal domain (domain I) is smaller than the C-terminal domain (domain II). Domain I comprises residues 1–107, which contains three α-helices (α1-3) and a four-stranded anti-parallel β-sheet in order β1-β4-β3-β2. Domain II comprises residues 112–271 that form five α-helices (α4–8), one short two-stranded parallel β-sheet (β5–β6) and one short two-stranded anti-parallel β-sheet (β7–β8). Residues 108–111 link the two domains. The PLP cofactor is covalently bound to Lys140 that is in domain II. The active site is formed in a cleft formed between the two domains (Figure 3).

Sixty residues from each subunit, provided by both domains, contribute to dimer formation. These residues participate in around 30 hydrogen-bonding associations and eight salt bridge interactions to stabilize the quaternary structure. Most of the residues that contribute to the dimer interface are found in two patches; residues Ala10 to Gly25 centred on α1 and residues Arg127 to Ala152 centered on α5.

The approximate dimensions of the dimer, which constitutes the asymmetric unit, are 80 Å×40 Å×40 Å and this displays a solvent accessible surface area of approximately 21,000 Å^2^. The contact area between the two subunits occupies approximately 2,050 Å^2^, about 15% of the surface of a subunit. These values are similar to those observed for other PabC proteins, for example the interface of the *E. coli* protein (PDB code 1et0) covers an area of 2,130 Å^2^.

The structures of PabC from three organisms (*E. coli*, *L. pneumophila* and *T. thermophilus*) display a high degree of structural conservation with *Pa*PabC. Least-squares fitting of the Cα positions of single subunits results in RMSD values of between 1.27 and 1.95 Å, with a coverage in the range of 81 to 92% of the structures. The sequence identity shared with *Pa*PabC, falls in the range 26% to 35%.

### The active site and mechanistic considerations

The active site of *Pa*PabC is positioned between the two domains of the monomer close to the dimer interface (Figure 3). Here, the PLP cofactor is covalently linked to Lys140, bound and oriented by interactions with key residues, a selection of which are depicted in Figure 2. The PLP phosphate accepts hydrogen bonds donated by a cluster of main chain amide groups from Val200, Met201 and Ser237, and the side chain of Arg46. The side chain of Arg46 is itself positioned by interactions with the side chain of Glu28, which in turn accepts a hydrogen bond from His43 ND1. Two well ordered water molecules mediate hydrogen bonding networks linking the phosphate group to Asn178 OD1 and Arg202 N on one side, the main chain O and N groups of Thr29 on the other. The cofactor ring is sandwiched between Val197 and the main chain of a tripeptide segment comprising Val175-Phe176-Ser177. A hydrogen bond between Asn236 ND2 and the carbonyl of Phe176 in conjunction with hydrogen bonds formed between the amide groups and the side chain of Glu161 helps to determine the conformation of the tripeptide that forms a tight turn, between β9 and β10, that covers part of the cofactor.

The PLP methyl group makes van der Waals interactions with Gln147 CG and is positioned 3.2 Å distant from the carbonyl oxygen of Val175. The geometry of this latter contact is compatible with the presence of a C-H•••O hydrogen bond [39]. The side chain of Gln147 is held in place by a hydrogen bond with Arg144 NE. The arginine side chain is itself positioned by hydrogen bonds formed with the carbonyl groups of Leu139 and His141. There are also three water molecules (data not shown), which form a complex network of hydrogen bonds linking the side chain of Arg144 to other sections of the enzyme. In addition the positive dipole from α6, the closest residue on this helix is Gly199, provides an attractive force to interact with the PLP phosphate. The side chain of Glu173 displays a degree of conformational freedom with two rotamers being observed. One rotamer places the side chain to accept a hydrogen bond donated from PLP N1 (Figure 2). The alternative rotamer places the carboxylate at a distance of 3.7 Å from N1 (data not shown). Tyr92 from the partner subunit forms a hydrogen bond to PLP O3. The cofactor is also likely to posses an intra-molecular hydrogen bond involving O3 and NZ of Lys140. The side chain of Phe27, Val197 and aliphatic components of Arg46 and Arg144 surround the side chain of Lys140.

No structures of PabC-ternary complexes have yet been published to inform on aspects of substrate recognition and enzyme activity. Given the importance of such complexes we judged it important to get such data and attempted to determine structures of *Pa*PabC in complex with a number of compounds, for example, the sample used for structure determination was crystallized in the presence of 10 mM PABA. However, we have been unable to identify electron density compatible with binding of any such ligand in the substrate-binding pocket. We judged therefore that the position of PLP and Lys140 NZ, together with the conservation of amino acid sequence and structures give an indication of where substrate binds and which residues are important for substrate recognition and catalytic function. Consideration of an amino acid sequence alignment and structural overlay of *Pa*PabC with three other structures in the PDB proved particularly informative. To this we added molecular modelling and docking of the covalent intermediate formed during catalysis.

**Figure 5:**
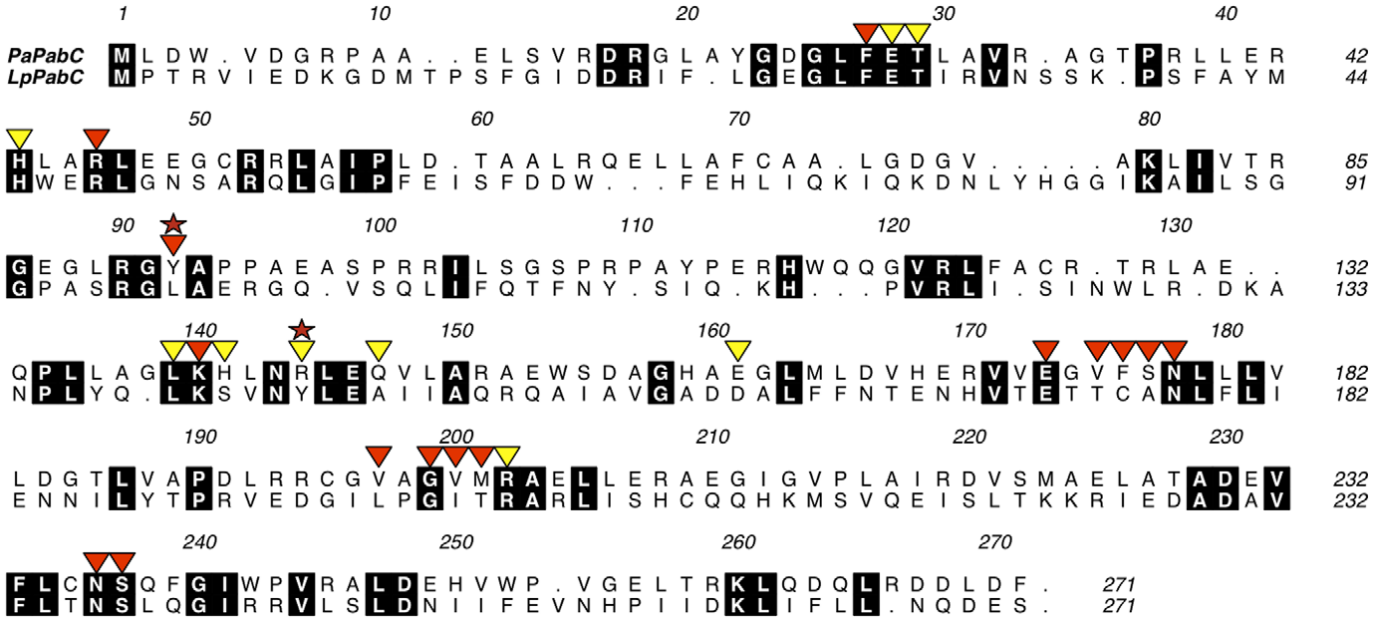
Structure-based alignment of *Pa*PabC and *Lp*PabC. Residues encased in black are strictly conserved. Red stars indicate the tyrosine residues at Position I (*Pa*PabC Y92) and Position II (*Lp*PabC Y144). Triangles mark residues that are discussed in the text. Red triangles identify Lys140 and residues that interact directly with the Lys140-PLP adduct; yellow triangles mark residues that contribute to the organization of the active site or that participate in solvent mediated interactions between the protein and the cofactor.

The contributions of 24 residues, within about 5 Å of PLP, to binding the cofactor and organising the active site are described above and summarized in Figure 5. Seven of these residues are strictly conserved in PabC of *P. aeruginosa*, *E. coli*, *L. pneumophila* and *T. thermophilus* (Phe27, Thr29, His43, Arg46, Gly199, Lys140 and Arg202 in *Pa*PabC). With one exception these residues contribute to the active site using the chemical properties of their side chains. The exception is Gly199 where any alteration on Cα would result in a steric clash with the phosphate group of PLP. Another seven residues contribute to the active site primarily through the hydrogen bonding capacity of the main chain (Val175, Phe176, Ser177, Leu139, His141, Val200 and Met201). Phe176 is not conserved in any of the three other sequences but the remainder are conserved in at least one other orthologue. Ser237 interacts with the PLP phosphate using main chain and side chain groups. This residue is only different in the *E. coli* sequence where an alanine is observed and an additional water molecule binds nearby helping to satisfy hydrogen-bonding capacity (data not shown). A similar observation is made when considering *Pa*PabC Asn236. The residue is conserved in *Ec*PabC and *Lp*PabC but changed to glycine in *Tt*PabC where a water molecule is observed to replace the side chain (data not shown). Asn178 is strictly conserved in *Ec*PabC and *Lp*PabC but changed to serine in *Tt*PabC. The side chain here simply helps to form one side of the cofactor-binding site. Val197 is conserved in *Ec*PabC and conservatively substituted as leucine in the other two sequences. The residue at this position in the active site forms van der Waals interactions with the cofactor. In *Pa*PabC Arg144 NE donates hydrogen bonds to Gln147 OE1 and the carbonyl of His141; interactions that help to create one side of the cofactor-binding site and to fix the position of Val197. These residues and the interactions they form appear to be conserved in *Ec*PabC but not in *Lp*PabC or *Tt*PabC. Sequence and structure alignments (not shown) indicate that *Pa*PabC Arg144 is replaced by tyrosine in both *Lp*PabC and *Tt*PabC and *Pa*PabC Gln147 replaced by an alanine in *Lp*PabC and a tyrosine in *Tt*PabC. This is an intriguing observation to be discussed below.

There are three glutamates amongst the 24 residues discussed above, Glu28, Glu161 and Glu173. Glu28 helps to position Arg46 to bind the cofactor phosphate. Both residues are strictly conserved in *Lp*PabC. A conservative substitution of the glutamate to threonine occurs in both *Ec*Pabc and *Tt*PabC and the hydrogen bonding interactions to support the role of an arginine binding the cofactor are preserved. Glu161 helps to configure a tripeptide segment to cover part of the cofactor and this feature is conserved since a glutamate is present in the other structures or, as in *Lp*PabC an aspartate occupies this position. The carboxylate side chain of Glu173 can interact directly with the cofactor by accepting a hydrogen bond donated from N1. In a similar fashion to what is observed for Glu161, such interactions occur in the orthologues with the residue either strictly conserved, or as in *Tt*PabC, replaced by aspartate.

A catalytic mechanism, based on previous studies of PLP-dependent enzymes, was proposed for PabC prior to any structural data being available [10]. Following structure determination of *Ec*PabC, a model of substrate binding was prepared to inform an assessment of the mechanism [15]. The contribution of PLP was considered the same, that is, following covalent linkage the enzyme-cofactor adduct undergoes a nucleophilic attack by the substrate amino group to produce a reactive external aldimine. This species then undergoes α-proton elimination, at C4, to the ε-amino group of a lysine to yield a reaction intermediate. In *Ec*PabC Lys159 forms the covalent link to the cofactor and likely responsible for α-proton abstraction. Nakai *et al.*, [15] further suggested that Thr28 of *Ec*PabC supplies a proton to reduce the olefin moiety of the substrate as pyruvate is released. To recover the Schiff-base form of the enzyme, PABA is released and PLP reacts with Lys159.

Three residues described as contributing to the mechanism of action in *Ec*PabC, Thr28, Lys97 and Lys159 [15], are strictly conserved in *Pa*PabC as Thr29, Lys80 and Lys140. They are also conserved in *Lp*PabC and *Tt*PabC with the conservative change of Lys80 to arginine in the thermophilic orthologue (Figure 5). Lys140 of *Pa*PabC is suitably placed to support α-proton abstraction and although Thr29 may engage in the proposed proton shuffling with both the olefin moiety of the substrate and Lys80 we noted an alternative residue that might fulfil this role. This residue may have been overlooked because Nakai *et al.*, [15] had access to only a single structure of PabC and alignments based on sequences alone would not identify a tyrosine that is conserved in three-dimensions.

**Figure 6:**
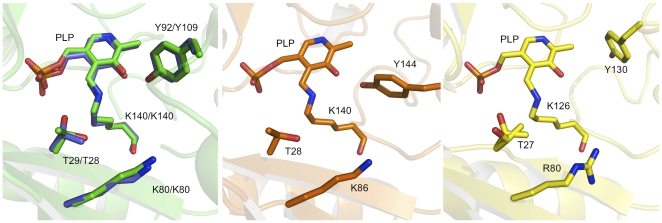
Structural conservation of tyrosine in the PabC active site. PLP is shown in the same manner as Figure 2. Left panel. A structural overlay of *Pa*PabC (green C atoms) and *Ec*PabC (blue C atoms). Here, Tyr92 and Tyr109, which represent position I, are contributed from the partner subunit. Centre panel. *Lp*PabC. Right panel. *Tt*PabC. Residue Thr27 displays two rotamers. In the *Lp*PabC and *Tt*PabC structures, Tyr144 and Tyr130 respectively represent position II and belong to the same subunit that forms the PLP-binding site.

In *Ec*PabC and *Pa*PabC tyrosine OH groups (Tyr109 and Tyr92 respectively), from the partner subunit, donate a hydrogen bond to PLP O3 (Figures 2, 6); an interaction that influences the electronic structure of the cofactor [15]. The sequence alignment of PabC orthologues suggests a degree of variation in the identity of the amino acid that occupies the position of this tyrosine (Figure 5). The structural overlays are striking since they clearly identify a conserved tyrosine in relation to the cofactor (Figure 6). In *Ec*PabC and *Pa*PabC, Tyr109 and Tyr92 respectively, are contributed from the partner subunit. We call this position I. However, in *Lp*PabC and *Tt*PabC, Tyr144 and Tyr130 respectively, are contributed from the same subunit, or position II.

We isolated 129 putative PabC sequences to investigate the distribution of tyrosine at these two positions seeking to address the hypothesis that if tyrosine is present at position I then it is likely absent from position II and *vice versa*. This hypothesis holds true for 125 out of the 129 sequences (data not shown). Two sequences, which share less than 30% sequence identity, have a tyrosine at both positions. Analyses of the sequences suggests that PabC can be classified into two groups depending on whether this active site tyrosine is provided by the same subunit which primarily forms the active site or from a partner subunit.

**Figure 7:**
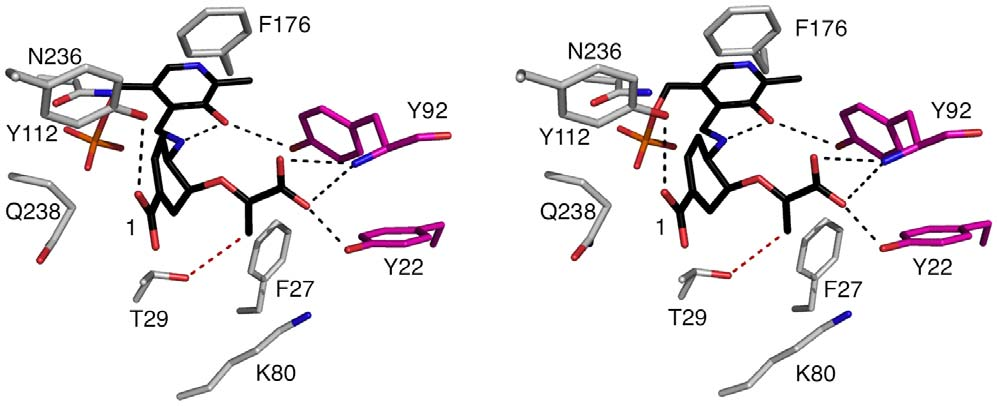
A stereoview of the catalytic intermediate docked into the active site of *Pa*PabC. The protein components are colored as in Figure 2. The C atoms of the modelled intermediate are black. Black dashed lines represent potential hydrogen bonding interactions, with a separation of 3.5 Å or less, and the red dashed line between Thr29 OG1 and the *sp* ^2^ hybridized C indicates the proximity of functional groups likely involved in catalysis.

Furthermore the high degree of conservation of an active site tyrosine in three-dimensional structures of PabC suggests a functional role. One possibility is that this residue could act as a proton donor for the methylene to methyl reduction that completes the reaction. Such a reduction would have little influence on the cleavage of the C-O bond of the substrate since the driving force for that reaction would be production of a conjugated aromatic six-carbon ring system. Our model of the catalytic intermediate (Figure 7) was specifically created to investigate the potential role of this active site tyrosine and to re-evaluate the reduction process that completes the reaction.

The model suggests that Tyr112 donates a hydrogen bond to the C1 carboxylate whilst, with contributions from the partner subunit, the main chain amide of Tyr92 and hydroxyl group of Tyr22 donate hydrogen bonds to the other carboxylate of the intermediate (Figure 7). Tyr92 contributes to the positioning and electronic structure of the cofactor by virtue of the hydrogen bond involving the hydroxyl group with the PLP carbonyl (Figure 2). The bulk of the tyrosine side chain serves to create a hydrophobic wall on one side of the active site. Although placed to interact with the catalytic intermediate this residue does not appear to be involved in donating a proton to convert methylene to methyl as part of the catalytic cycle (Figure 7). Rather, Thr29, as first postulated by Nakai *et al.*, [15] appears to fulfil such a role with the hydroxyl group placed about 3.5 Å from the olefin (Figure 7). Lys80 NZ is around 4.3 Å distant from the *sp* ^2^ hybridized C and it is possible that an alternative rotamer could bring this functional group into play with respect to either providing a proton itself or in activating the Thr29 hydroxyl for proton donation. We note that Thr29 is strictly conserved in 123 out of the 129 PabC sequences and conservatively substituted by serine in another five sequences. The single sequence in which we could not identify conservation in terms of a threonine or serine corresponding to Thr29 is the putative PabC from *Pantoea vagans*. This is a poorly conserved sequence sharing approximately 30% identity.

To further investigate the structure-activity relationship of PabC, and to assess the potential of this enzyme as a target for structure-based drug discovery will require the powerful combination of steady-state kinetic analysis with site-directed mutagenesis studies, and to carry out screening of chemical libraries. It will be imperative to extend from our work and to elucidate a means whereby structural data on enzyme-ligand complexes can be obtained.

## References

[1] Ye Q, Liu J, Walsh CT (1990). *p*-Aminobenzoate synthesis in *Escherichia coli*: Purification and characterization of PabB as aminodeoxychorismate synthase and enzyme X as aminodeoxychorismate lyase.. Proc Natl Acad Sci U S A.

[2] Green JM, Nichols BP (1991). p-Aminobenzoate biosynthesis in *Escherichia coli*.. J Biol Chem.

[3] Nichols BP, Seibold AM, Doktor SZ (1989). para-Aminobenzoate synthesis from chorismate occurs in two steps.. J Biol Chem.

[4] Anderson KS, Kati WM, Ye Q, Liu J, Walsh CT (1991). Isolation and structure elucidation of the 4-amino-4-deoxychorismate intermediate in the PABA enzymatic pathway.. J Am Chem Soc.

[5] Parsons JF, Jensen PY, Pachikara AS, Howard AJ, Eisenstein E (2002). Structure of *Escherichia coli* aminodeoxychorismate synthase: architectural conservation and diversity in chorismate-utilizing enzymes.. Biochemistry.

[6] Slock J, Stahly DP, Han CY, Six EW, Crawford IP (1990). An apparent *Bacillus subtilis* folic acid biosynthetic operon containing pab, an amphibolic trpG gene, a third gene required for the synthesis of para-aminobenzoic acid, and the dihydropteroate synthase gene.. J Bacteriol.

[7] Schadt HS, Schadt S, Oldach F, Sussmuth RD (2009). 2-Amino-2-deoxychorismate is a key intermediate in *Bacillus subtilis* p-aminobenzoic acid biosynthesis.. J Am Chem Soc.

[8] Aparicio JF, Caffrey P, Gil JA, Zotchev SB (2003). Polyene antibiotic biosynthesis gene clusters.. Appl Microbiol Biotechnol.

[9] Zhang Y, Bai L, Deng Z (2009). Functional characterization of the first two actinomycete 4-amino-4-deoxychorismate lyase genes.. Microbiol.

[10] Green JM, Merkel WK, Nichols BP (1992). Characterization and sequence of Escherichia coli pabC, the gene encoding aminodeoxychorismate lyase, a pyridoxal phosphate-containing enzyme.. J Bacteriol.

[11] Hoang TT, Karkhoff-Schweizer RR, Kutchma AJ, Schweizer HP (1998). A broad-host-range Flp-FRT recombination system for site-specific excision of chromosomally-located DNA sequences: application for isolation of unmarked *Pseudomonas aeruginosa* mutants.. Gene.

[12] Salama NR, Shepherd B, Falkow S (2004). Global transposon mutagenesis and essential gene analysis of *Helicobacter pylori*.. J Bacteriol.

[13] de Berardinis V, Vallenet D, Castelli V, Besnard M, Pinet A (2008). A complete collection of single-gene deletion mutants of *Acinetobacter baylyi* ADP1.. Mol Syst Biol.

[14] Brown GM (1962). The biosynthesis of folic acid. II. Inhibition by sulfonamides.. J Biol Chem.

[15] Nakai T, Mizutani H, Miyahara I, Hirotsu K, Takeda S (2000). Three-dimensional structure of 4-amino-4-deoxychorismate lyase from *Escherichia coli*.. J Biochem.

[16] Padmanabhan B, Bessho Y, Ebihara A, Antonyuk SV, Ellis MJ (2009). Structure of putative 4-amino-4-deoxychorismate lyase from *Thermus thermophilus* HB8.. Acta Crystallogr F.

[17] Eliot AC, Kirsch JF (2004). Pyridoxal phosphate enzymes: Mechanistic, structural, and evolutionary considerations.. Annu Rev Biochem.

[18] Jhee K, Yoshimura T, Miles EW, Takeda S, Miyahara I (2000). Stereochemistry of the transamination reaction catalyzed by aminodeoxychorismate lyase from *Escherichia coli*: Close relationship between fold type and stereochemistry.. J Biochem.

[19] Soper TS, Manning JM (1981). Different modes of action of inhibitors of bacterial D-amino acid transaminase. A target enzyme for the design of new antibacterial agents.. J Biol Chem.

[20] Castell A, Mille C, Unge T (2010). Structural analysis of mycobacterial branched-chain aminotransferase: implications for inhibitor design.. Acta Cryst D.

[21] Hu LY, Boxer PA, Kesten SR, Lei HJ, Wustrow DJ (2006). The design and synthesis of human branched-chain amino acid aminotransferase inhibitors for treatment of neurodegenerative diseases.. Bioorg Med Chem Lett.

[22] Lepore BW, Liu D, Peng Y, Fu M, Yasuda C (2010). Chiral discrimination among aminotransferases: Inactivation by 4-amino-4,5-dihydrothiophenecarboxylic acid.. Biochem.

[23] Hunter WN (2009). Structure-based ligand design and the promise held for antiprotozoan drug discovery.. J Biol Chem.

[24] George AM, Jones PM, Middleton PG (2009). Cystic fibrosis infections: treatment strategies and prospects.. FEMS Microbiol Lett.

[25] Kerr KG, Snelling AM (2009). *Pseudomonas aeruginosa*: a formidable and ever-present adversary.. J Hosp Infect.

[26] Gasteiger E, Hoogland C, Gattiker A, Duvaud S, Wilkins MR, Walker JM (2005). Protein Identification and Analysis Tools on the ExPASy Server.. The Proteomics Protocols Handbook.

[27] Leslie AG (2006). The integration of macromolecular diffraction data.. Acta Crystallogr D.

[28] Evans P (2006). Scaling and assessment of data quality.. Acta Crystallogr D.

[29] Vagin A, Teplyakov A (1997). MOLREP: an automated program for molecular replacement.. J Appl Crystallogr.

[30] Murshudov GN, Vagin AA, Dodson EJ (1997). Refinement of macromolecular structures by the maximum-likelihood method.. Acta Crystallogr D.

[31] Collaborative Computational Project, Number 4 (1994). Acta Cryst D.

[32] Emsley P, Cowtan K (2004). *Coot*: model-building tools molecular graphics.. Acta Crystallogr D.

[33] Davis IW, Leaver-Fay A, Chen VB, Block JN, Kapral GJ (2007). MolProbity: all-atom contacts and structure validation for proteins and nucleic acids.. Nucleic Acids Res.

[34] Krissinel E, Henrick K (2007). Inference of macromolecular assemblies from crystalline state.. J Mol Biol.

[35] Bond CS, Schuttelkopf AW (2009). ALINE: a WYSIWYG protein-sequence alignment editor for publication-quality alignments.. Acta Cryst.

[36] DeLano WL (2002).

[37] Edgar RC (2004). MUSCLE: multiple sequence alignment with high accuracy and high throughput.. Nucleic Acids Res.

[38] Schüttelkopf AW, van Aalten DM (2004). PRODRG: a tool for high-throughput crystallography of protein-ligand complexes.. Acta Crystallogr D.

[39] Leonard GA, McAuley-Hecht K, Brown T, Hunter WN (1995). Do C-H•••O hydrogen bonds contribute to the stability of nucleic acid base pairs?. Acta Crystallogr D.

